# Atomic-level structure of the amorphous drug atuliflapon *via* NMR crystallography†

**DOI:** 10.1039/d4fd00078a

**Published:** 2024-07-17

**Authors:** Jacob B. Holmes, Daria Torodii, Martins Balodis, Manuel Cordova, Albert Hofstetter, Federico Paruzzo, Sten O. Nilsson Lill, Emma Eriksson, Pierrick Berruyer, Bruno Simões de Almeida, Mike Quayle, Stefan Norberg, Anna Svensk Ankarberg, Staffan Schantz, Lyndon Emsley

**Affiliations:** a Institut des Sciences et Ingénierie Chimiques, Ecole Polytechnique Fédérale de Lausanne (EPFL) CH-1015 Lausanne Switzerland lyndon.emsley@epfl.ch; b National Centre for Computational Design and Discovery of Novel Materials MARVEL, Ecole Polytechnique Fédérale de Lausanne (EPFL) CH-1015 Lausanne Switzerland; c Data Science & Modelling, Pharmaceutical Sciences, R&D, AstraZeneca Gothenburg Sweden; d Oral Product Development, Pharmaceutical Technology & Development, Operations, AstraZeneca Gothenburg Sweden

## Abstract

We determine the complete atomic-level structure of the amorphous form of the drug atuliflapon, a 5-lipooxygenase activating protein (FLAP) inhibitor, *via* chemical-shift-driven NMR crystallography. The ensemble of preferred structures allows us to identify a number of specific conformations and interactions that stabilize the amorphous structure. These include preferred hydrogen-bonding motifs with water and with other drug molecules, as well as conformations of the cyclohexane and pyrazole rings that stabilize structure by indirectly allowing for optimization of hydrogen bonding.

## Abstract 2

We determine the complete atomic-level structure of the amorphous form of the drug atuliflapon, a 5-lipooxygenase activating protein (FLAP) inhibitor, *via* chemical-shift-driven NMR crystallography.

## Graphical abstract

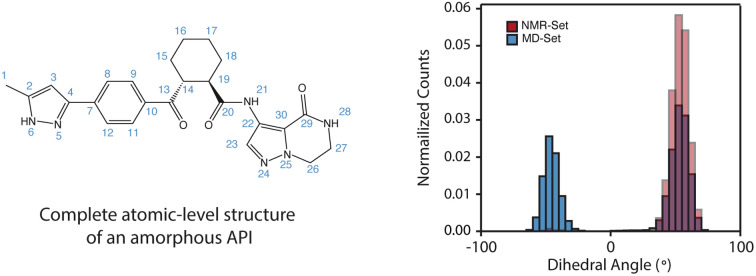

## 1 Introduction

Amorphous solids are of high interest in pharmaceutical formulations as they can have increased solubility compared with crystalline formulations, which opens the chemical landscape available during the drug discovery step.^1–4^ While amorphous formulations are promising, they are prone to recrystallization which limits their applicability. To identify the key stabilizing interactions of amorphous forms, the structure and chemical environment need to be characterized at the atomic level.

The nature of disordered solids means that no single conformation or atomic environment can be used to describe the structure, and instead a set of chemical environments is needed to adequately describe the experimental ensemble. This disorder limits the structural information that can be obtained from typical structure determination techniques such as X-ray diffraction,^5–14^ absorption fine structure,^15^ electron diffraction,^16–18^ electron microscopy,^19,20^ and nuclear magnetic resonance.^21^

In contrast, it has recently been shown that NMR spectroscopy can be used to fully characterize amorphous molecular solids at the atomic level.^22,23^ NMR is sensitive to the local chemical environment and is therefore not subject to the requirement of long-range order.^21,24–26^ NMR crystallography methods are versatile, and have been applied to molecular systems ranging from molecular solids^27–30^ to enzyme active sites,^31–33^ passivating layers in photovoltaic materials,^34–36^ and cements,^37–39^ resulting in a complete chemical structure being determined.

The workflow for complete structure determination by chemical-shift-driven NMR crystallography consists in comparing computed or predicted chemical shifts for candidate structures to those observed in experiments, and ranking structures based on their agreement. In amorphous solids, it requires millions of candidate structures to find both molecular conformations and surrounding environments that are consistent with the observed NMR shifts, and this scales exponentially as the conformational degrees of freedom and intermolecular hydrogen-bonding interactions increase. For amorphous solids, NMR crystallography is able to identify sets of structures that highlight promoted conformations and intermolecular interactions.

In NMR spectra, the chemical disorder associated with amorphous solids manifests as peak broadening with linewidths an order of magnitude larger than for microcrystalline organics.^40,41^ This broadening is what captures the structural distributions, and instead of an atom being assigned to a single chemical-shift value, it is assigned to a distribution that is determined experimentally. Candidate structures are generated using molecular dynamics simulations to capture the largest possible landscape of possible chemical environments (referred to in the following as the MD set). Recently enabled by machine learning methods,^42,43^ chemical shifts can today be predicted for millions of structures within a few days with accuracy similar to that of DFT. These predicted shifts are then compared to the experimental distributions where a small (*e.g.*, 5009) subset of the structures in best agreement with the data corresponds to the experimentally determined preferred structures (which compose what we refer to as the NMR set).

Previously, this methodology was first applied to the drug molecule atuliflapon (or AZD5718)^22^ to understand the change in mean chemical shift observed for a single hydrogen-bonded ^1^H atom between the crystalline and amorphous form. It was shown how hydrogen bonding to water plays a critical role in mechanisms of stability for these solids.^22,23^ The first complete structure determination of an amorphous solid was then carried out for AZD4625,^23^ determined by using all the assigned chemical shifts.

Here we expand on the previous work on amorphous atuliflapon, a 5-lipooxygenase activating protein (FLAP) inhibitor,^22^ to provide a complete determination of the chemical structure using all of the assigned ^1^H and ^13^C chemical shifts. The MD set comprises nearly 3 million chemical environments, and we are able to characterize the preferred conformational space described in the set of 5009 structures in best agreement with the NMR chemical-shift distributions. We find that the cyclohexane ring is held in the chair conformation with both of the large attached functional groups in the equatorial position. From three-dimensional average atomic density plots, we observe that O13 and O20 preferentially point in opposite directions, despite O20 having very little selection in the NMR set as compared to the MD set. To improve the statistical significance of our observations, we use a local approach to characterizing the hydrogen-bonding interactions of H6 where only the heavy atoms and corresponding hydrogens within 3 bonds are used for the selection of environments. This highlights the use of partial assignments for structure determination, which is a valuable insight as molecules become larger and more complex.

## 2 Methods

### 2.1 MD simulations

The MD simulations used here have been reported previously.^22^ As described previously, the GROMACS program (version 2016.4)^44^ was used for all MD simulations. The systems were initially equilibrated for 1 ns using the canonical (NVT) ensemble at 298 K. A second equilibration was carried out for 10 ns using an isothermal–isobaric ensemble (NPT) at 298 K and 1 bar. Production simulations were carried out for 600 ns using the NPT ensemble at 298 K and 1 bar, where the temperature and pressure were held constant. A particle mesh Ewald scheme^45^ was used to compute the electrostatic interactions with a 10 Å cutoff for the real space. The same cutoff was used for van der Waals interactions, with long-range dispersion correction applied to both energy and pressure. Bond lengths to hydrogens were constrained using the LINCS algorithm.^46^ Models of the amorphous structure were obtained by extracting 1001 evenly spaced snapshots from the last 100 ns of each MD simulation, corresponding to 100 ps time steps between the extracted snapshots. Further details, and a link to all the raw data, can be found in ref. 22.

### 2.2 Chemical-shift predictions

The molecular environments were constructed with a central molecule and all molecules with at least one atom within 7 Å of the central molecule. The chemical shifts of these environments were predicted using ShiftML2.^42^ To convert from shielding to shift, offsets of 30.78 and 170.04 for ^1^H and ^13^C were used, respectively.^23^

### 2.3 Calculation of formation energies

The formation energies of local molecular environments were computed as described in ref. 23. The environments were defined as described above but with a cutoff distance of 7 Å. The difference in energy between the environments with and without the central molecule provides both the conformational energy of the central molecule and the energy of intermolecular interactions with the environment. The energies were computed using the DFTB-D3H5 semiempirical level of theory using the 3ob-3-1 parameter set and the DFTB+ software version 24.^47–53^

The energies of all NMR selected molecular environments were computed and compared to the energies of a set of 2200 randomly selected molecular environments from the MD set (100 environments selected per MD run).

### 2.4 Three-dimensional atomic density maps

To visualize the atomic environments, average density maps^54^ were constructed as described in ref. 54 for the NMR set and for a subset of randomly selected environments from the MD set. Gaussian functions with a width of 0.5 Å were placed at all atomic positions and evaluated on a cubic grid with 12 Å sides, centered on the first aligned atom. The Gaussians were normalized such that if an atom is present in each environment at the same point in space, then the maximum intensity at this point in space is one.

### 2.5 NMR assignment

**Fig. 1:**
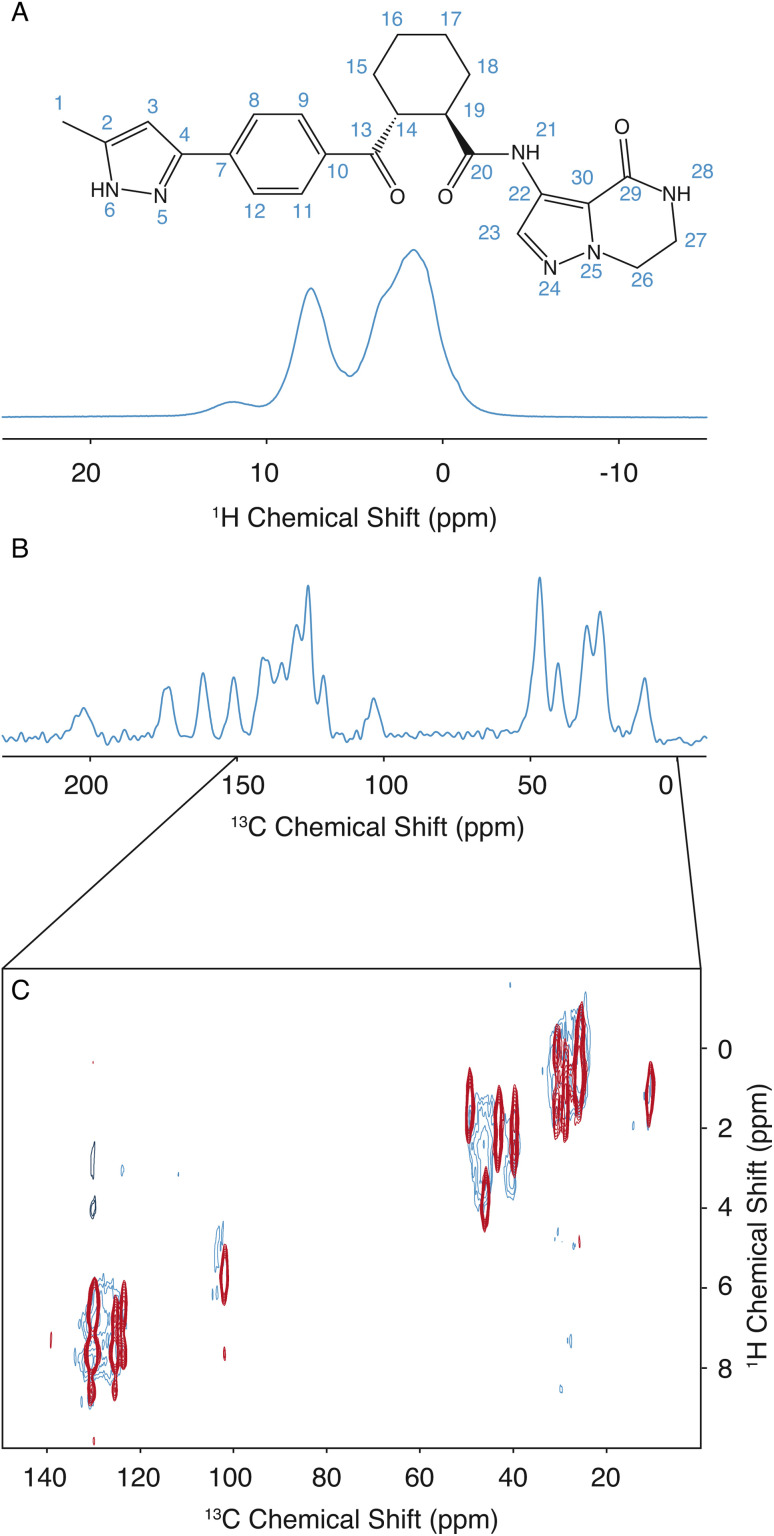
The 2D structure of atuliflapon is shown inset. (A) 900 MHz 1D ^1^H spectrum of amorphous atuliflapon acquired at 298 K with an MAS rate of 62.5 kHz. (B) 125 MHz 1D ^13^C CPMAS spectrum acquired at 298 K with a MAS rate of 22 kHz. (C) 2D ^1^H–^13^C HETCOR spectra collected for the crystalline (red/black) and amorphous (blue) forms at 298 K with a MAS rate of 22 kHz at a ^13^C Larmor frequency of 125 MHz.

The assignment for the crystalline form of atuliflapon has been previously determined,^22^ and these assignments were then used as starting points for the assignment of the amorphous formulation here. The remarkable overlap of the ^13^C CPMAS spectra of the crystalline and amorphous forms, and the ^1^H–^13^C HETCOR, shown in Fig. 1C, made for a straightforward assignment of the amorphous form here. The crystalline assignment was used as a starting point to fit Gaussian distributions in the ^13^C CPMAS spectrum of the amorphous form, with the peaks being assigned to the mean chemical shift closest to the assigned crystalline shifts. Using the mean position of the now assigned amorphous peaks, the ^1^H–^13^C HETCOR was used to determine Gaussian distributions for the ^1^H distributions for any correlation peaks observed at the respective mean ^13^C chemical shift. The full list of assigned distributions obtained in this way is given in the ESI† together with the experimental details used to obtain the spectra. (Note that here we simplify the analysis by assuming the chemical-shift distributions are all Gaussian, but in principle the experimentally measured distributions can also be used directly.)

## 3 Results and discussion

The assignment of the amorphous form was determined as described above. Then, to determine the set of structures promoted by NMR, the same methodology as used by Cordova *et al.* for AZD4625 (ref. 23) is employed here. A pool of 2.8 million molecules with diverse conformations and molecular environments was obtained from MD simulations. For each ^1^H and ^13^C site in each of the molecules in the MD snapshots, the chemical shift was predicted using ShiftML2,^43,55^ and local molecular environments were obtained from the MD simulations as described above. The predicted shifts for a molecule in a given local molecular environment were then used to calculate a global probability that the environment matches the NMR data.^23^ The histogram of probabilities for all the molecular environments to match the NMR experiments is shown in Fig. S2.† The NMR set was constructed by selecting structures with a global probability above *p* = 0.237, representing the top 0.18% (5009 out of 2 818 816 molecular environments) of structures that best match the NMR data. This NMR structure can now be analyzed to determined preferred conformations and hydrogen-bonding patterns in order to determine the mechanisms that stabilize the amorphous form.

### 3.1 Hydrogen bonding

**Fig. 2:**
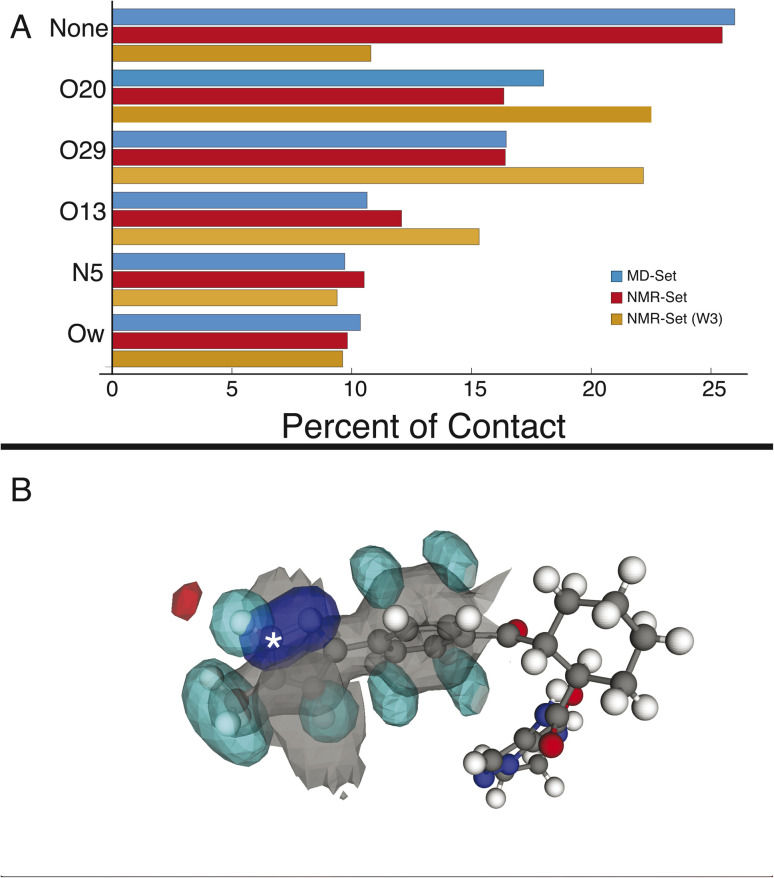
(A) Percentage of hydrogen-bond occurrences with H6 in the MD set (blue), the NMR set using all shifts (red), and the NMR set using shifts occurring within 3 bonds (orange). (B) Three-dimensional atomic density map aligned on N6 (indicated with an asterisk) showing the average density for the NMR set. The preferred H-bonding interaction between an oxygen and NH6 is clearly seen top left. The colors gray, blue, teal, and red represent carbon, nitrogen, hydrogen, and oxygen density, respectively.

We first examine the hydrogen-bonding partners of H6, which was the focus of the previous study,^22^ by comparing the MD set to the NMR set in Fig. 2. Atuliflapon has 6 potential hydrogen-bond acceptors, O13, O20, O29, N5, N24 and N25, with water providing another. Using all the assigned chemical shifts, we see no specific promotion of hydrogen-bonding motifs in the NMR set as compared to the background MD set, which is against chemical intuition. The mean hydrogen chemical shift for H6 is 11.9 ppm, which strongly suggests a preferred hydrogen-bonding interaction.^56,57^ To examine this interaction in detail, we use a subset of the chemical shifts to evaluate the local structure, specifically corresponding to the carbon atoms within 3 bonds of N6 and their attached hydrogen atoms, resulting in 8 out of 46 chemical shifts being used (W3 in the notation of Cordova *et al.*^58^), to improve specificity for H6. We see a stark change, shown in Fig. 2, where environments with hydrogen-bonding interactions are now strongly promoted, and the environments without a hydrogen-bond acceptor are demoted. The contrast in results stems from allowing all assigned chemical shifts to weigh equally for all portions of the molecule, or in favoring more local shifts. With this in mind, we suggest that conformational changes beyond 5 Å have little impact on the chemical shift unless an intramolecular interaction is being formed. In the case of N6, there is no evidence of intramolecular hydrogen bonding, so the local approach is justified. We further justify this by comparing the *p*-values for the best 5175 structures. In the W3 selection, the minimum *p*-value for the best structures is 0.441 and for the selection including all atoms, the minimum *p*-value is 0.237. The range of minimum *p*-values with a W3 selection for the other hydrogen-bond acceptors and donors is 0.322–0.390. Using this local approach, we observe that the intermolecular hydrogen bonding to O13, O20, and O29 is strongly promoted to 22.51%, 22.18%, and 15.32% in the NMR set, compared with 18.01%,16.45%, and 10.64% in the MD set. We see the demotion of no interactions from 25.99% to 10.80% in the MD set and NMR set, respectively. Within the NMR set, the formation energy decreases by at least 5 kJ mol^−1^ in environments where a hydrogen bond is formed.

### 3.2 Cyclohexane ring

**Fig. 3:**
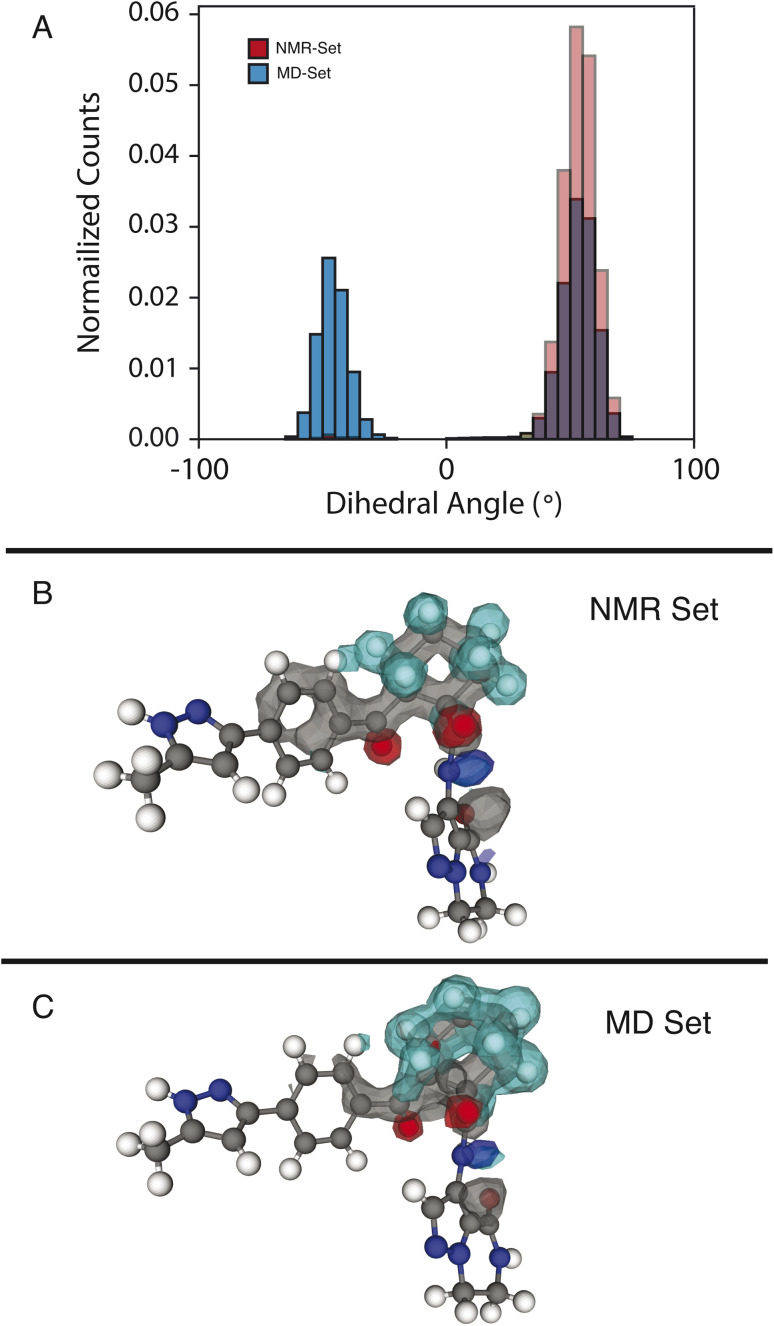
(A) Histogram of dihedral angles for the C14–C19 bond in the MD set (blue) and NMR set with all shifts (red). (B and C) Three-dimensional atomic density maps aligned about the C14–C19 bond showing the density for the NMR set (B) and the MD set (C). The colors gray, blue, teal, and red represent carbon, nitrogen, hydrogen, and oxygen, respectively.

We examine two dihedral angles in the cyclohexane ring to identify promoted molecular conformations in the NMR set. In Fig. 3, we clearly see a strong promotion for the chair conformation, which places the large functional groups connected to C14 and C19 in the equatorial position along the ring. In the NMR set, the formation energies are ∼15 kJ mol^−1^ lower than those for similar dihedral angles in the MD set. We note that the selection for the NMR set is guided only by the chemical-shift distributions and does not consider energy. This additional stabilization likely comes from the ability that this ring conformation lends to form hydrogen bonds to neighboring molecules. In the three-dimensional atomic density map^54^ shown in Fig. 3B, O13 and O20 are positioned in opposite directions, which might explain the overall stabilization due to the chair conformations.

In the MD set, we see a significant carbon density observed above C19 (in the cyclohexane ring), with oxygen density above the ring, which is reduced in the NMR set, indicating a reduced presence of the axial orientation in the NMR set. This is an interesting observation as C20 itself shows very little selection of the chemical shifts in the NMR set (Fig. S4†), but a change in position is observed in the atomic density maps. This highlights that the positions of individual atoms are experimentally determined by an ensemble of the local chemical shifts.

### 3.3 Pyrazole ring orientation

**Fig. 4:**
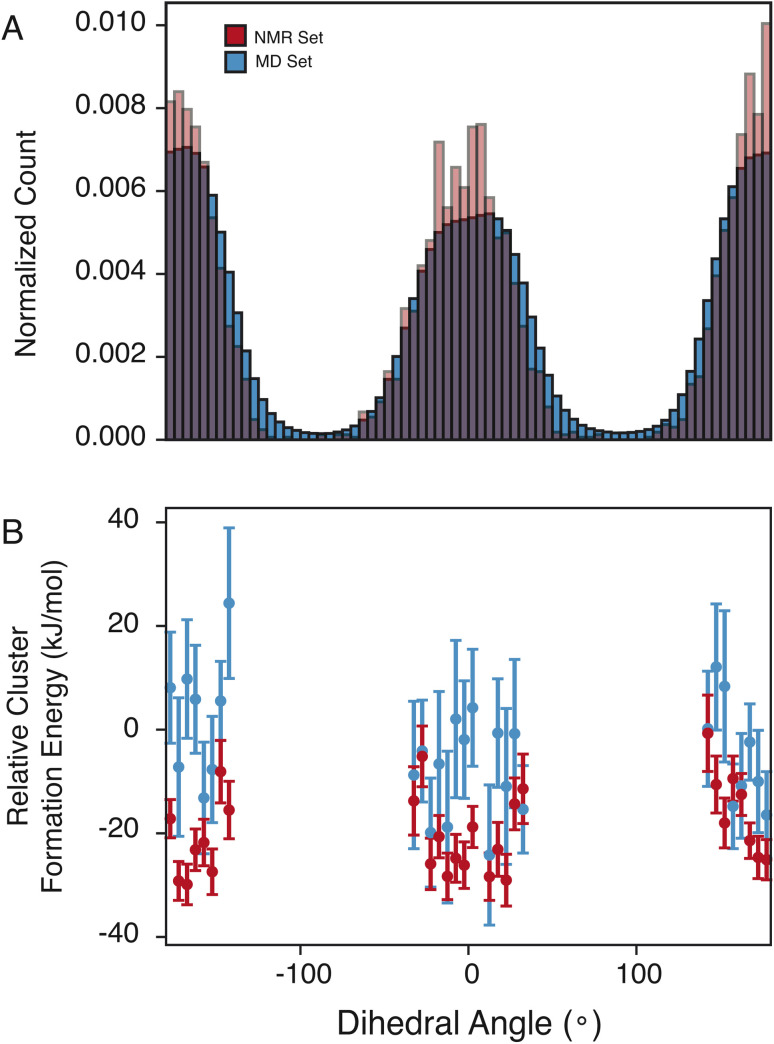
(A) Histogram of the dihedral angles for the C4–C7 bond in the MD set (blue) and NMR set with all shifts in (red). (B) Relative cluster energy as a function of dihedral angle for a random selection of the MD set (blue) and the NMR set (red).

The pyrazole ring contains N6, which has a diverse set of hydrogen-bond acceptors as discussed above and shown in Fig. 2, and likely samples orientations that can accommodate hydrogen bonding. In Fig. 4, we examine the torsion angle of the pyrazole ring with respect to the benzyl group. In the NMR set, there is clear promotion of dihedral angles centered about 0 and 180°, with demotion of conformations centered around −90 and 90°. These promoted regions are stabilized by up to 30 kJ mol^−1^ compared to random selections of the MD set. While the promoted conformations are abundant in both the MD set and the NMR set, it is important to note again that the NMR set is only driven by the agreement with the experimental chemical shifts. However, we see consistently that the energy of the NMR set is lower than that of the random selection of the MD set. This drives a hypothesis that conformational changes that are themselves low in energy can play a crucial role in stabilizing other more beneficial interactions such as hydrogen bonding.

## 4 Conclusion

We have determined the complete atomic-level structure of the amorphous form of the drug atuliflapon *via* chemical-shift-driven NMR crystallography. The ensemble of preferred structures determined using NMR allows us to identify a number of conformations and interactions that stabilize the amorphous structure. Specifically, H6 is found to interact with water molecules, and to have promoted intermolecular interactions with the carbonyl groups. The stabilization is reflected in the energies being at least 5 kJ mol^−1^ lower for environments forming hydrogen bonds. The cyclohexane ring remains largely in the chair conformation, positioning O20 and O13 in opposite directions, allowing H21 to orient close to O13. Lastly, the pyrazole ring has a clearly preferred orientation, which is stabilized by ∼10 kJ mol^−1^, likely reflecting adjustments in the conformation so that H6 can form hydrogen bonds.

## Conflicts of interest

The AstraZeneca authors disclose that they are employees of AstraZeneca and that they have ownership, options, or interests in AstraZeneca stock.

## Supplementary Material

FD-255-D4FD00078A-s001

## References

[cit1] Babu N. J., Nangia A. (2011). Solubility Advantage of Amorphous Drugs and Pharmaceutical Cocrystals. Cryst. Growth Des..

[cit2] Kawabata Y., Wada K., Nakatani M., Yamada S., Onoue S. (2011). Formulation design for poorly water-soluble drugs based on biopharmaceutics classification system: Basic approaches and practical applications. Int. J. Pharm..

[cit3] Laitinen R., Löbmann K., Strachan C. J., Grohganz H., Rades T. (2013). Emerging trends in the stabilization of amorphous drugs. Int. J. Pharm..

[cit4] Yu L. (2001). Amorphous pharmaceutical solids: preparation, characterization and stabilization. Adv. Drug Delivery Rev..

[cit5] Egami T. and Billinge S. J., Underneath the Bragg Peaks: Structural Analysis of Complex Materials, Elsevier, 2003

[cit6] Gemmi M. (2019). *et al.*, 3D electron diffraction: the nanocrystallography revolution. ACS Cent. Sci..

[cit7] Gruene T., Holstein J. J., Clever G. H., Keppler B. (2021). Establishing electron diffraction in chemical crystallography. Nat. Rev. Chem.

[cit8] Harris K. D. , Powder Diffraction Crystallography of Molecular Solids, Advanced X-Ray Crystallography, 2012, pp. 133–177 10.1007/128_2011_251 21952843

[cit9] Huang Z., Grape E. S., Li J., Inge A. K., Zou X. (2021). 3D electron diffraction as an important technique for structure elucidation of metal-organic frameworks and covalent organic frameworks. Coord. Chem. Rev..

[cit10] Hughes C. E., Boughdiri I., Bouakkaz C., Williams P. A., Harris K. D. (2018). Elucidating the crystal structure of dl-arginine by combined powder X-ray diffraction data analysis and periodic DFT-D calculations. Cryst. Growth Des..

[cit11] Jones C. G. (2018). *et al*.**, The CryoEM Method MicroED as a Powerful Tool for Small Molecule Structure Determination. ACS Cent. Sci..

[cit12] Kelton K. F. (2003). *et al*.**, First X-Ray Scattering Studies on Electrostatically Levitated Metallic Liquids: Demonstrated Influence of Local Icosahedral Order on the Nucleation Barrier. Phys. Rev. Lett..

[cit13] Nannenga B. L., Gonen T. (2019). The cryo-EM method microcrystal electron diffraction (MicroED). Nat. Methods.

[cit14] Rietveld H. M. (1969). A profile refinement method for nuclear and magnetic structures. J. Appl. Crystallogr..

[cit15] Sheng H. W., Luo W. K., Alamgir F. M., Bai J. M., Ma E. (2006). Atomic packing and short-to-medium-range order in metallic glasses. Nature.

[cit16] Hirata A. (2011). *et al.*, Direct observation of local atomic order in a metallic glass. Nat. Mater..

[cit17] Hirata A. (2013). *et al.*, Geometric Frustration of Icosahedron in Metallic Glasses. Science.

[cit18] Pekin T. C. (2019). *et al.*, Direct measurement of nanostructural change during *in situ* deformation of a bulk metallic glass. Nat. Commun..

[cit19] Hwang J. (2012). *et al.*, Nanoscale structure and structural relaxation in Zr_50_ Cu_45_ Al_5_ bulk metallic glass. Phys. Rev. Lett..

[cit20] Zhong L., Wang J., Sheng H., Zhang Z., Mao S. X. (2014). Formation of monatomic metallic glasses through ultrafast liquid quenching. Nature.

[cit21] Hodgkinson P. (2020). NMR crystallography of molecular organics. Prog. Nucl. Magn. Reson. Spectrosc..

[cit22] Cordova M. (2021). *et al.*, Structure determination of an amorphous drug through large-scale NMR predictions. Nat. Commun..

[cit23] Cordova M. (2023). *et al.*, Atomic-level structure determination of amorphous molecular solids by NMR. Nat. Commun..

[cit24] Ashbrook S. E., McKay D. (2016). Combining solid-state NMR spectroscopywith
first-principles calculations - a guide to NMR crystallography. Chem. Commun..

[cit25] Moran R. F., Dawson D. M., Ashbrook S. E. (2017). Exploiting NMR spectroscopy for the study of disorder in solids. Int. Rev. Phys. Chem..

[cit26] Reif B., Ashbrook S. E., Emsley L., Hong M. (2021). Solid-state NMR spectroscopy. Nat. Rev. Methods Primers.

[cit27] Baias M. (2013). *et al.*, De novo determination of the crystal structure of a large drug molecule by crystal structure prediction-based powder NMR crystallography. J. Am. Chem. Soc..

[cit28] Baias M. (2013). *et al.*, Powder crystallography of pharmaceutical materials by combined crystal structure prediction and solid-state 1H NMR spectroscopy. Phys. Chem. Chem. Phys..

[cit29] Balodis M., Cordova M., Hofstetter A., Day G. M., Emsley L. (2022). De Novo Crystal Structure Determination from Machine Learned Chemical Shifts. J. Am. Chem. Soc..

[cit30] Salager E. (2010). *et al.*, Powder crystallography by combined crystal structure prediction and high-resolution ^1^H solid-state NMR spectroscopy. J. Am. Chem. Soc..

[cit31] Bertarello A. (2020). *et al.*, Picometer Resolution Structure of the Coordination Sphere in the Metal-Binding Site in a Metalloprotein by NMR. J. Am. Chem. Soc..

[cit32] Holmes J. B. (2022). *et al.*, Imaging active site chemistry and protonation states: NMR crystallography of the tryptophan synthase α-aminoacrylate intermediate. Proc. Natl. Acad. Sci. U. S. A..

[cit33] Singh H. (2019). *et al.*, Fast Microsecond Dynamics of the Protein–Water Network in the Active Site of Human Carbonic Anhydrase II Studied by Solid-State NMR Spectroscopy. J. Am. Chem. Soc..

[cit34] Hope M. A. (2021). *et al.*, Nanoscale phase segregation in supramolecular π-templating for hybrid perovskite photovoltaics from NMR crystallography. J. Am. Chem. Soc..

[cit35] Kubicki D. J., Stranks S. D., Grey C. P., Emsley L. (2021). NMR spectroscopy probes microstructure, dynamics and doping of metal halide perovskites. Nat. Rev. Chem.

[cit36] Mishra A. (2022). *et al.*, Dynamic nuclear polarization enables NMR of surface passivating agents on hybrid perovskite thin films. J. Am. Chem. Soc..

[cit37] Kunhi Mohamed A. (2020). *et al.*, The atomic-level structure of cementitious calcium aluminate silicate hydrate. J. Am. Chem. Soc..

[cit38] Morales-Melgares A. (2022). *et al.*, Atomic-Level Structure of Zinc-Modified Cementitious Calcium Silicate Hydrate. J. Am. Chem. Soc..

[cit39] Walkley B., Provis J. (2019). Solid-state nuclear magnetic resonance spectroscopy of cements. Mater. Today Adv..

[cit40] Simões de Almeida B., Torodii D., Moutzouri P., Emsley L. (2023). Barriers to resolution in ^1^H NMR of rotating solids. J. Magn. Reson..

[cit41] Zorin V. E., Brown S. P., Hodgkinson P. (2006). Origins of linewidth in H^1^ magic-angle spinning NMR. J. Chem. Phys..

[cit42] Cordova M. (2022). *et al.*, A Machine Learning Model of Chemical Shifts for Chemically and Structurally Diverse Molecular Solids. J. Phys. Chem. C.

[cit43] Paruzzo F. M. (2018). *et al.*, Chemical shifts in molecular solids by machine learning. Nat. Commun..

[cit44] Van der Spoel D. (2005). *et al.*, GROMACS: fast, flexible, and free. J. Comput. Chem..

[cit45] Darden T., York D., Pedersen L. (1993). Particle Mesh Ewald - an N.Log(N) Method for Ewald Sums in Large Systems. J. Chem. Phys..

[cit46] Hess B. (2008). P-LINCS: a parallel linear constraint solver for molecular simulation. J. Chem. Theory Comput..

[cit47] Aradi B., Hourahine B., Frauenheim T. (2007). DFTB+, a sparse matrix-based implementation of the DFTB method. J. Phys.Chem. A.

[cit48] Elstner M. (1998). *et al.*, Self-consistent-charge density-functional tight-binding method for simulations of complex materials properties. Phys. Rev. B: Condens. Matter Mater. Phys..

[cit49] Gaus M., Cui Q., Elstner M. (2011). DFTB3: extension of the self-consistent-charge density-functional tight-binding method (SCC-DFTB). J. Chem. Theory Comput..

[cit50] Gaus M., Goez A., Elstner M. (2013). Parametrization and benchmark of DFTB3 for organic molecules. J. Chem. Theory Comput..

[cit51] Hourahine B. (2020). *et al.*, DFTB+, a software package for efficient approximate density functional theory based atomistic simulations. J. Chem. Phys..

[cit52] Rezac J. (2017). Empirical self-consistent correction for the description of hydrogen bonds in DFTB3. J. Chem. Theory Comput..

[cit53] Yang Y., Yu H., York D., Cui Q., Elstner M. (2007). Extension of the self-consistent-charge density-functional tight-binding method: third-order expansion of the density functional theory total energy and introduction of a modified effective coulomb interaction. J. Phys. Chem. A.

[cit54] Cordova M., Emsley L. (2023). Chemical Shift-Dependent Interaction Maps in Molecular Solids. J. Am. Chem. Soc..

[cit55] Cordova M. (2022). *et al.*, A machine learning model of chemical shifts for chemically and structurally diverse molecular solids. J. Phys. Chem. C.

[cit56] Chan-Huot M., Sharif S., Tolstoy P. M., Toney M. D., Limbach H. H. (2010). NMR Studies of the Stability, Protonation States, and Tautomerism of C- and N-Labeled Aldimines of the Coenzyme Pyridoxal 5′-Phosphate in Water. Biochem..

[cit57] Limbach H. H. (2011). *et al.*, Critical hydrogen bonds and protonation states of pyridoxal 5′-phosphate revealed by NMR. Biochim. Biophys. Acta, Proteins Proteomics.

[cit58] Cordova M., Balodis M., Simoes de Almeida B., Ceriotti M., Emsley L. (2021). Bayesian probabilistic assignment of chemical shifts in organic solids. Sci. Adv..

