# Supplementary material for: Atomic-level structure of the amorphous drug atuliflapon via NMR crystallography
Source: Faraday Discuss. 2024 Jul 17;255:342–54. doi: 10.1039/d4fd00078a (PMC11409164; doi:10.1039/d4fd00078a)
Supplement: FD-255-D4FD00078A-s001 [file FD-255-D4FD00078A-s001.pdf]

Supplementary Information (SI) for Faraday Discussions.
This journal is © The Royal Society of Chemistry 2024

Atomic-level structure of the amorphous drug Atuliflapon by NMR crystallography

Jacob B. Holmes[1,2], Daria Torodii[1], Martins Balodis[1], Manuel Cordova[1,2], Albert Hofstetter[1], Federico Paruzzo[1], Sten Nilsson Lill[3], Emma Eriksson[3], Pierrick Berruyer[1], Bruno Simões de Almeida[1], Mike Quayle[4], Stefan Norberg[4] Anna Svensk Ankarberg[4], Staffan Schantz[4]*, Lyndon Emsley[1,2]*

[1] Institut des Sciences et Ingénierie Chimiques, Ecole Polytechnique Fédérale de Lausanne (EPFL), CH-1015 Lausanne, Switzerland

[2] National Centre for Computational Design and Discovery of Novel Materials MARVEL, Ecole Polytechnique Fédérale de Lausanne (EPFL), CH-1015 Lausanne, Switzerland.

[3] Data Science & Modelling, Pharmaceutical Sciences, R&D, AstraZeneca, Gothenburg, Sweden

[4] Oral Product Development, Pharmaceutical Technology & Development, Operations, AstraZeneca, Gothenburg, Sweden.

Supporting Information

Raw data statement: All data and code used are available from https://doi.org/10.24435/materialscloud:9r-b9 under the license CC-BY-4.0 (Creative Commons Attribution-ShareAlike 4.0 International).

Table of Contents

1. Experimental Details 1
2. Molecular Dynamic Simulations 1
3. Experimental chemical shift distributions 1
4. Predicted chemical shift distributions 4
5. Relative Cluster Energies of molecular interactions and conformations 8

Experimental Details
All spectra used in this analysis have previously been published in ref [1] and can be referred to for more detail. The 1H spectrum was collected using a 0.7mm probe at a 1H larmor frequency of 900 MHz at 298K with an MAS rate of 62.5kHz. The 13C CPMAS and 1H-13C HETCOR spectra were collected using a 1.3 mm probe at a 1H larmor frequency of 500 MHz.

Molecular dynamics for the amorphous structures

The MD simulations used here have been reported previously and as described in ref[1], molecular dynamic (MD) simulations were carried out on periodic amorphous cells with 0, 0.5, 1, 2, and 4% (w/w) of water molecules. The atomic positions from the single crystal XRD structure were used as a starting point for optimization in the gas-phase as the B3LYP-D3/6-31G(d,p)[2-5] level of theory using Gaussian 09 revised D.01 program[6]. CHELPG and optimized coordinates were extracted from the DFT optimization and used to generate the amorphous cells. Cubic amorphous cells were created using Material Studio[7] and COMPASS-II[8] force fields with 128 molecules of AZ5718 within the cells. The OPLS_2005[9,10] force field parameters generated using Schrödinger ffld_server[11] with the optimized coordinates and CHELPG charges as inputs. The "ffconv.py" tool was used to convert the topology into GROMCS format[12]. Water was treated using the TIP3P model in the simulations[13].

Experimental chemical shift distributions
Table S1: Solid-State NMR assignment of ^{13}C Chemical shifts of the amorphous AZD5718

Atom Index	μ (ppm)	σ (ppm)
C1	11.38	1.73
C2	140.38	2.29
C3	103.36	1.93
C4	151.09	1.5
C7	140.38	2.29
C8	125.58	1.22
C9	129.73	1.89
C10	134.84	1.53
C11	129.73	1.89
C12	125.58	1.22
C13	202.66	2.81
C14	46.95	1.83
C15	30.78	1.88
C16	25.93	1.54
C17	25.93	1.54
C18	30.78	1.88
C19	46.95	1.83
C20	173.79	1.91
C22	125.58	1.22
C23	129.74	1.89

C26	46.95	1.83
C27	40.38	1.39
C29	161.41	1.91
C30	120.61	1.29

Table S1: Solid-State NMR assignment of ^{1}H Chemical shifts of the amorphous AZD5718

Atom Index	μ (ppm)	σ (ppm)
H1	1.56	0.55
H3	5.17	0.82
H6	11.84	2
H8	7.07	0.99
H9	7.11	0.98
H11	7.11	0.98
H12	7.07	0.99
H14	2.67	0.9
H15a	0.54	0.88
H15b	0.54	0.88
H16a	0.39	0.85
H16b	0.39	0.85
H17a	0.39	0.85
H17b	0.39	0.85
H18a	0.54	0.88
H18b	0.54	0.88
H19	2.67	0.9
H23	7.11	0.98
H26a	2.67	0.9
H26b	2.67	0.9
H27a	2.87	0.79
H27b	2.87	0.79

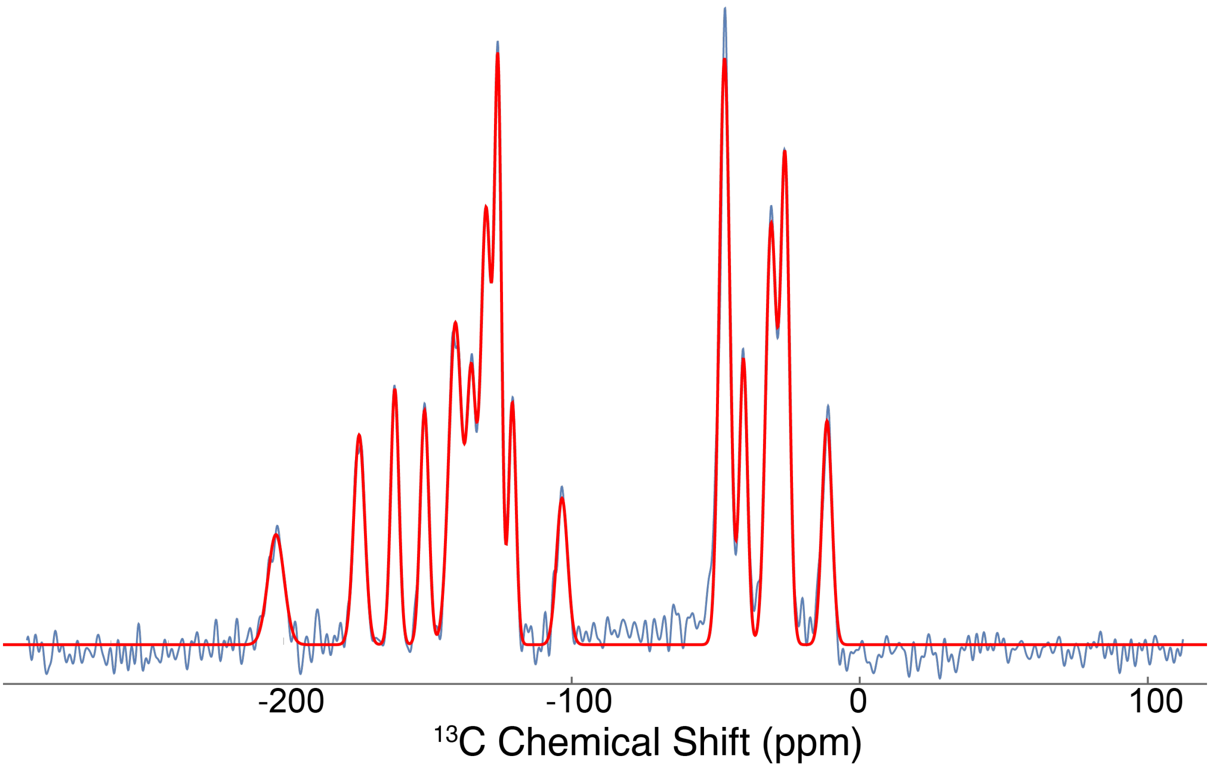

Figure S1: Peak fitting of the ^{13}C CPMAS spectrum (blue) with the Gaussian using the parameters listed in Table S1 (red)

Predicted chemical shift distributions

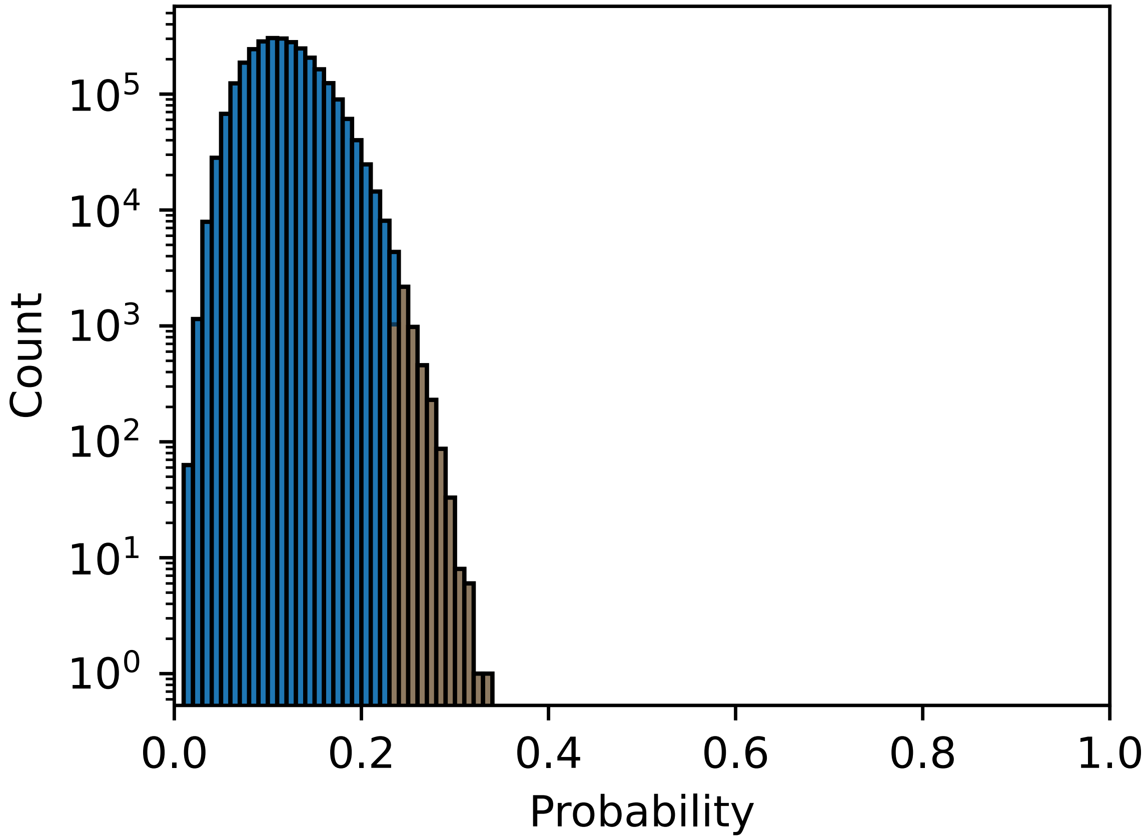

Figure S2: Histogram of p-values for each structure in the MD set (blue) and the NMR set (orange) for all shifts. The counts are shown on the log scale.

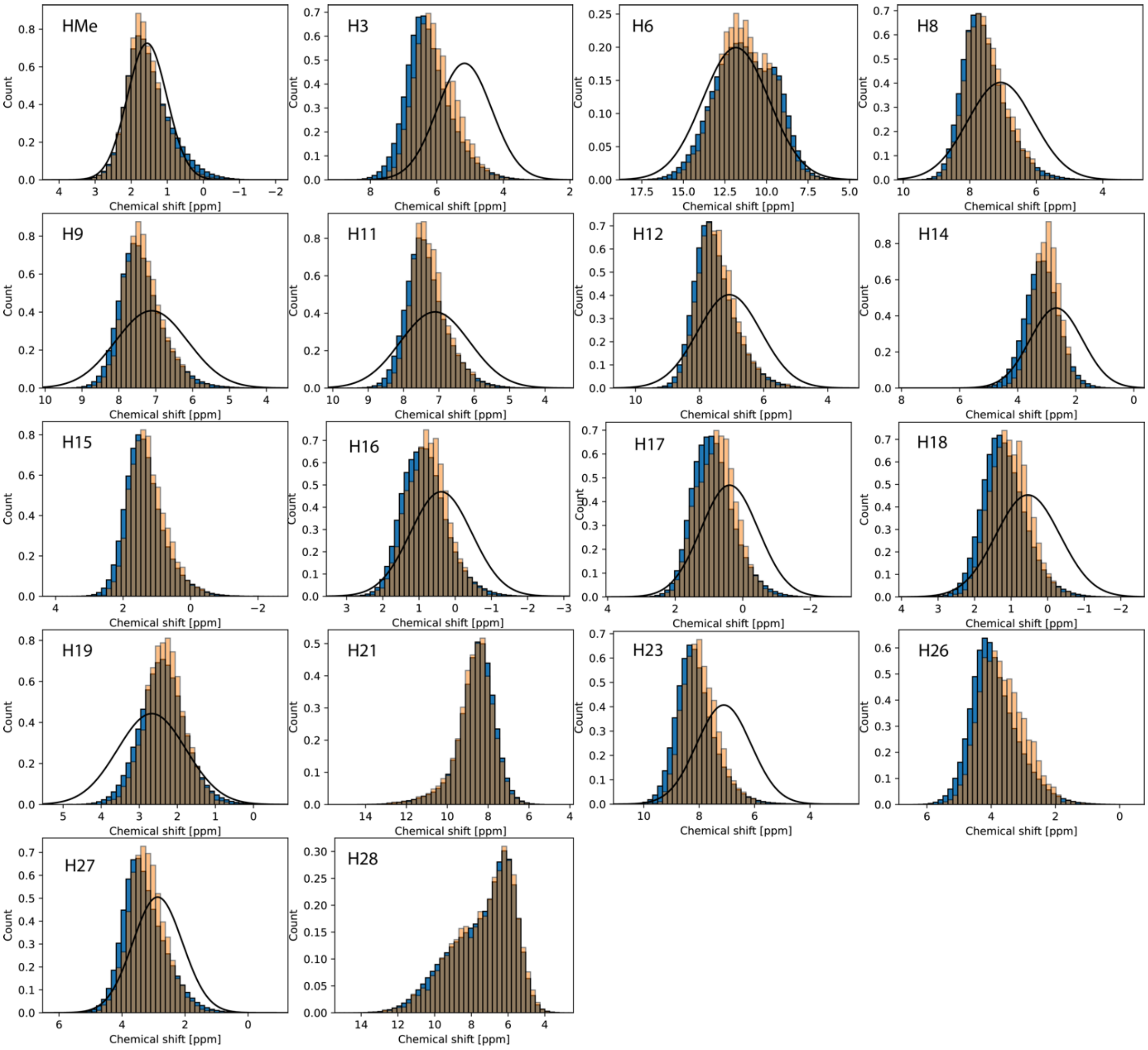

Figure S3: Histogram of predicted ^{1}H chemical shifts using shiftML2 for the MD set (orange) and the NMR set (orange) with the assigned experimental distribution shown in black.

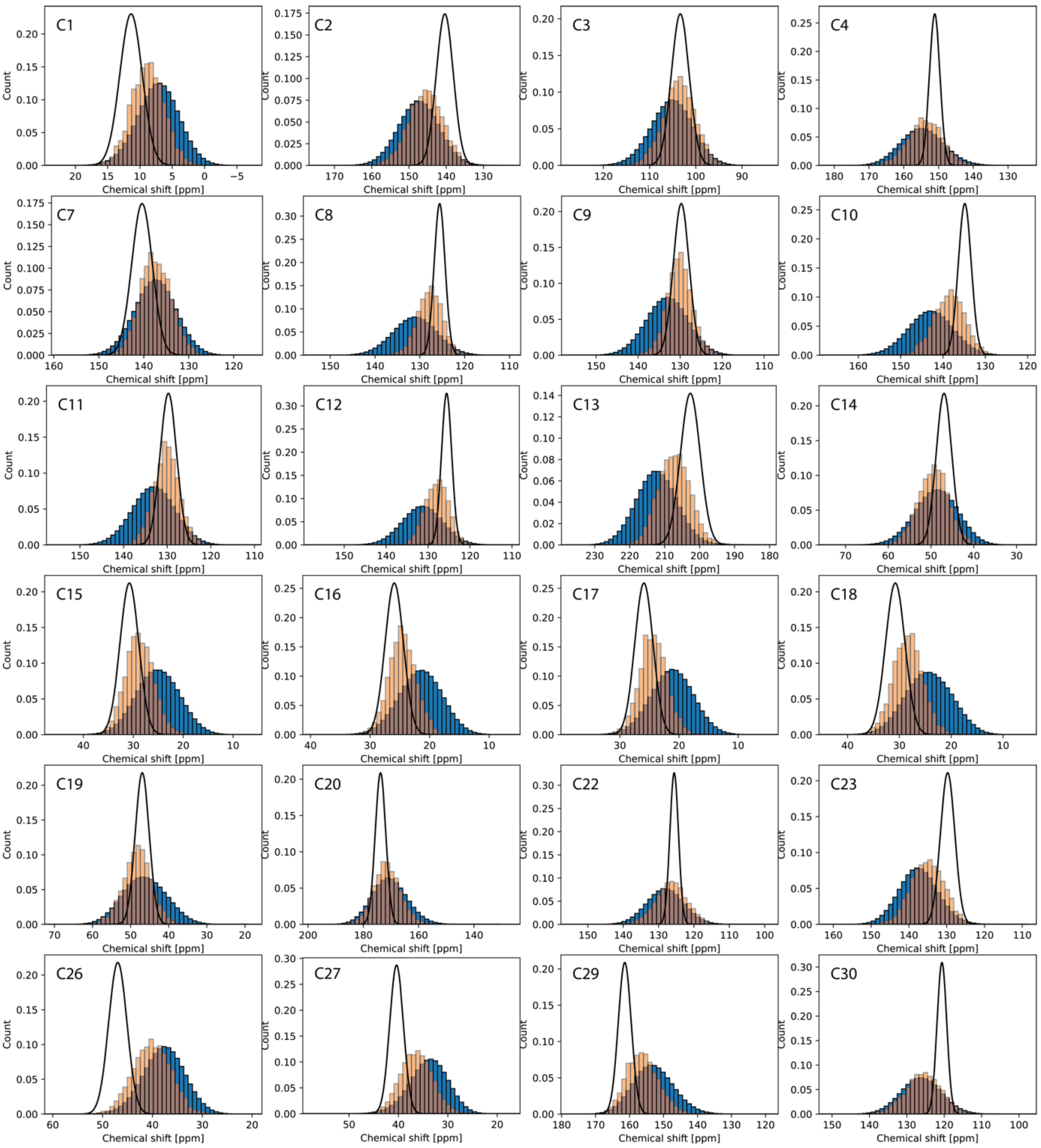

Figure S4: Histogram of predicted ^{13}C chemical shifts using shiftML2 for the MD set (orange) and the NMR set (orange) with the experimental distribution shown in black

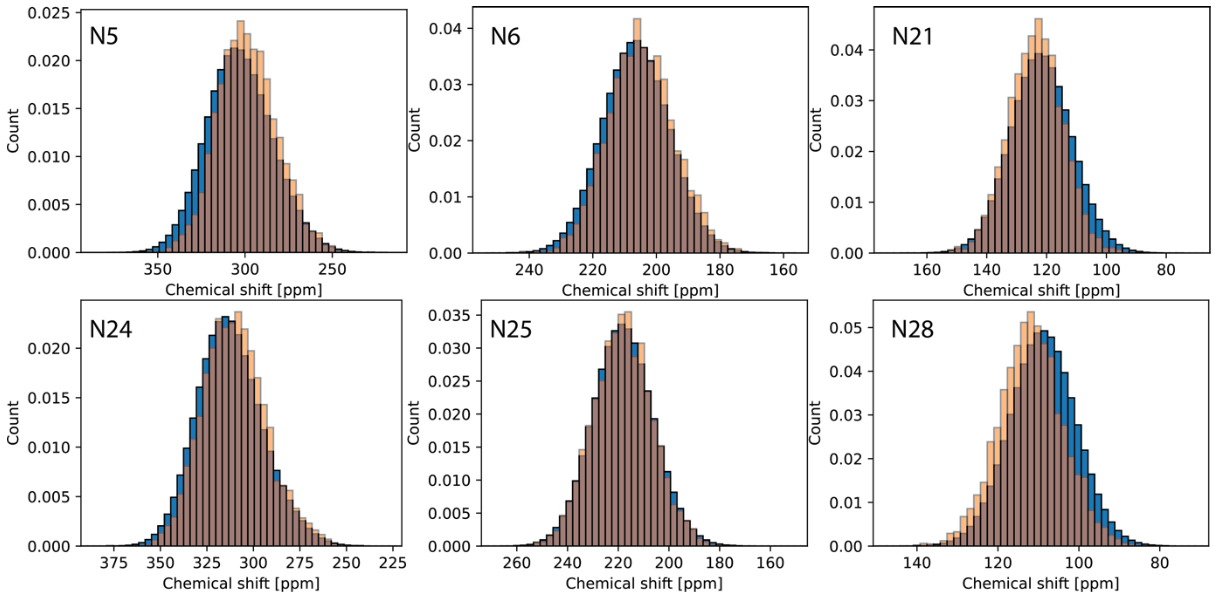

Figure S5: Histogram of predicted ^{15}N chemical shifts using shiftML2 for the MD set (orange) and the NMR set (orange)

Relative Cluster Energies of molecular interactions and conformations

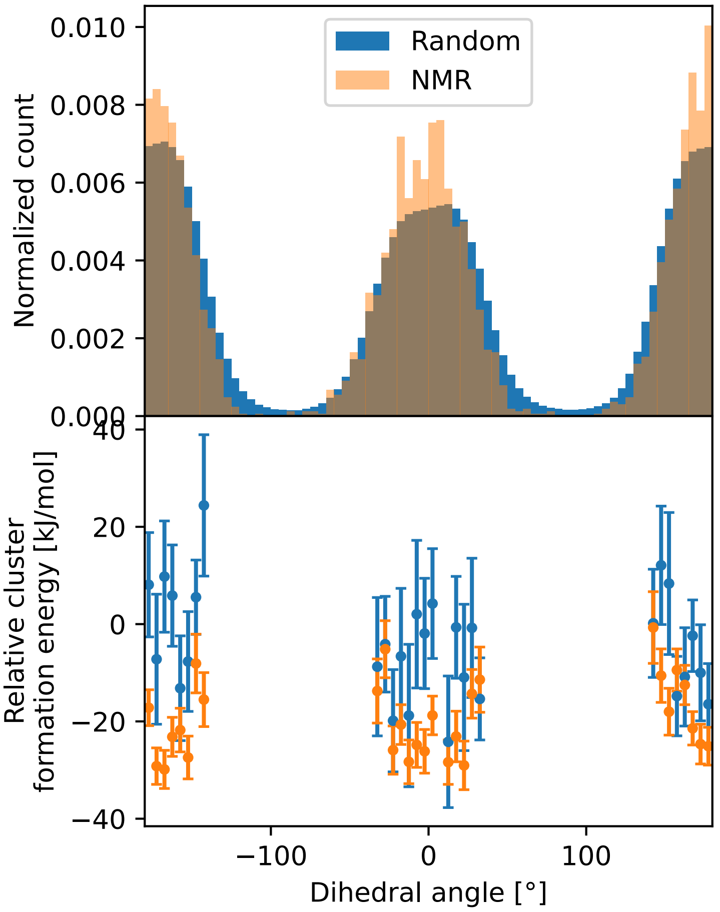

Figure S6: (top) histogram of torsion angle between C3-C4-C7-C8 for the MD set (blue) and the NMR set (orange). (bottom) Relative cluster energies as a function of dihedral angle for the NMR set (orange) and random selections of the MD set (orange)

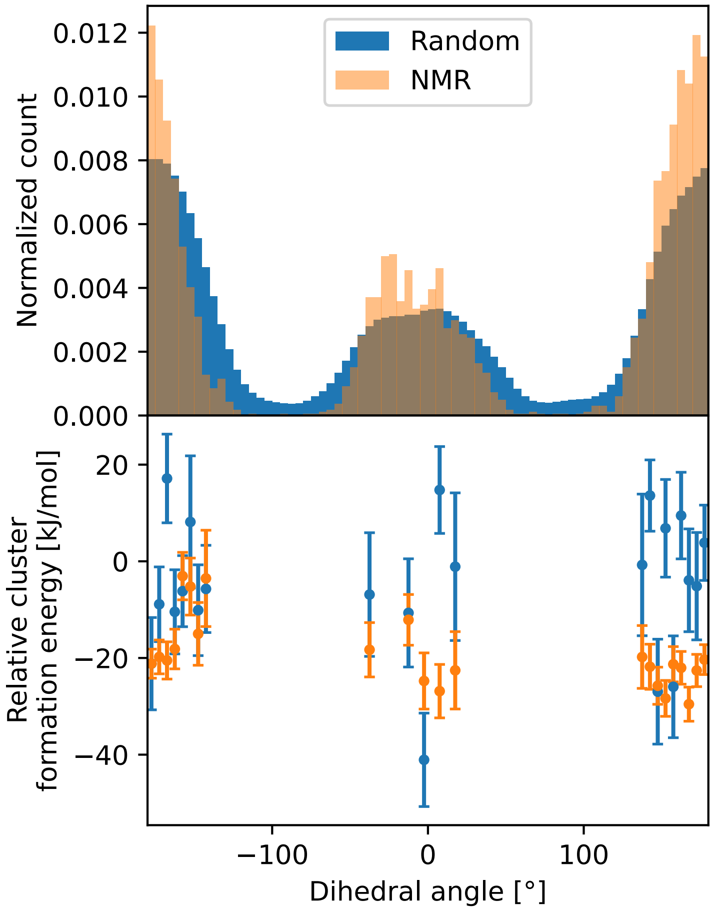

Figure S7: (top) histogram of torsion angle between C9-C10-C13-C14 for the MD set (blue) and the NMR set (orange). (bottom) Relative cluster energies as a function of dihedral angle for the NMR set (orange) and random selections of the MD set (orange)

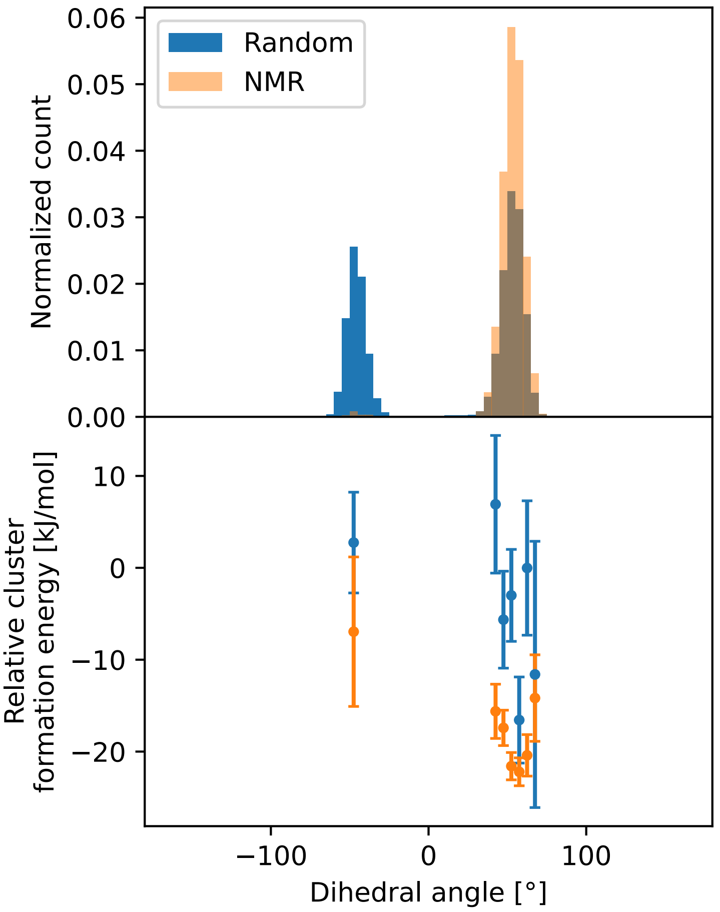

Figure S8: (top) histogram of torsion angle between C15-C14-C19-C18 for the MD set (blue) and the NMR set (orange). (bottom) Relative cluster energies as a function of dihedral angle for the NMR set (orange) and random selections of the MD set (orange)

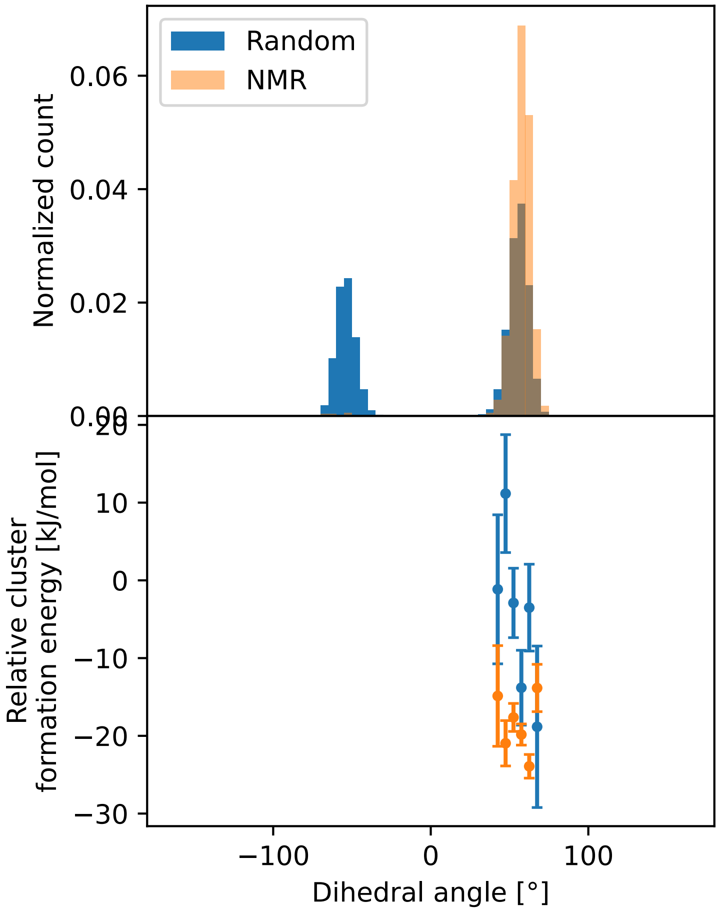

Figure S9: (top) histogram of torsion angle betweenC16-C17-C18-C19 for the MD set (blue) and the NMR set (orange). (bottom) Relative cluster energies as a function of dihedral angle for the NMR set (orange) and random selections of the MD set (orange)

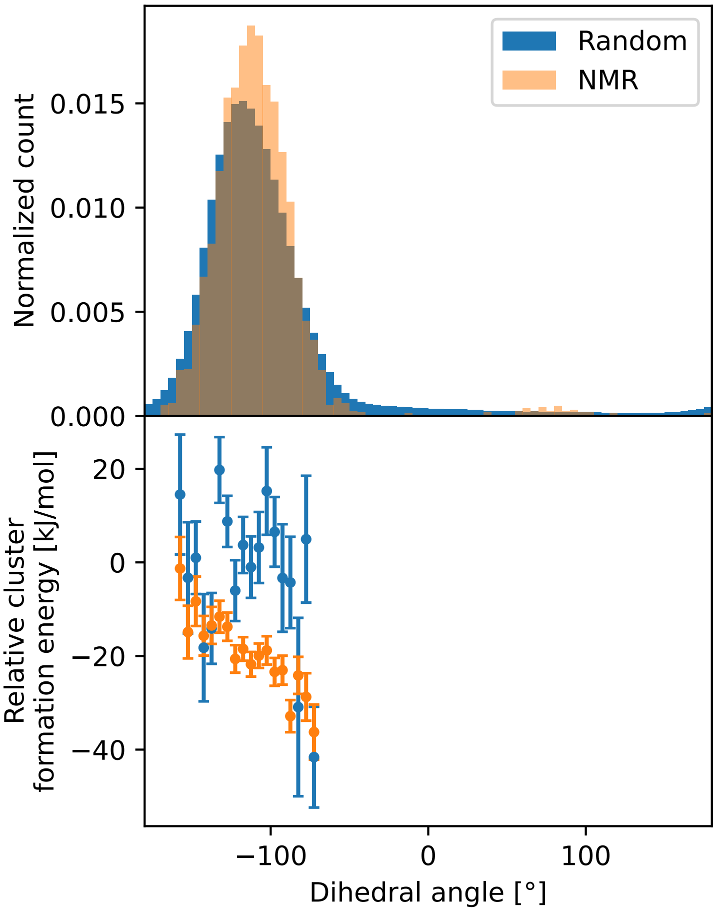

Figure S10: (top) histogram of torsion angle between N21-C20-C19-C18 for the MD set (blue) and the NMR set (orange). (bottom) Relative cluster energies as a function of dihedral angle for the NMR set (orange) and random selections of the MD set (orange)

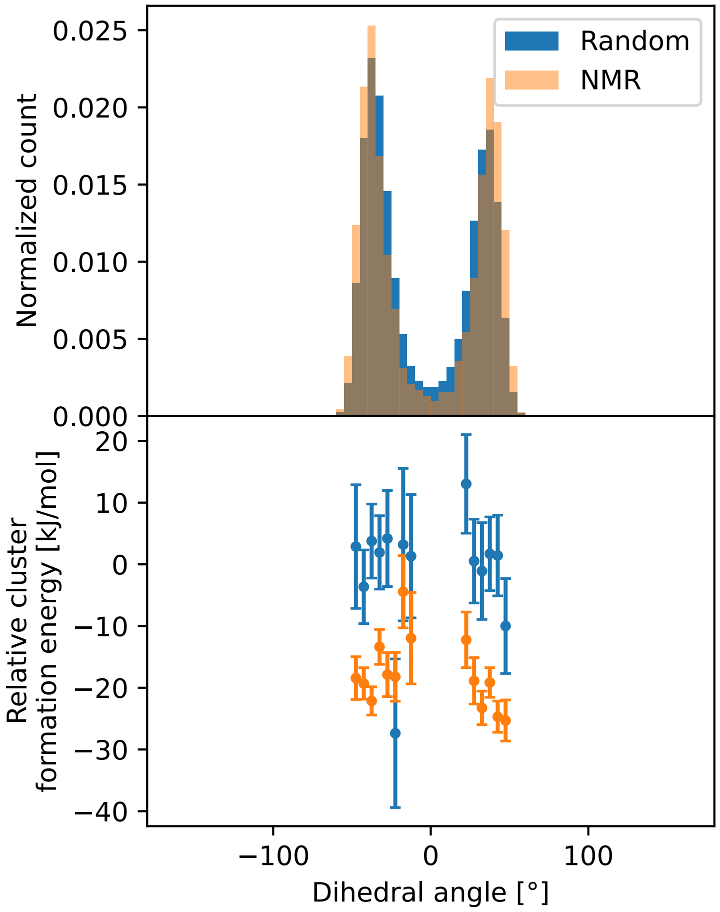

Figure S11: (top) histogram of torsion angle between N25-C26-C27-N28 for the MD set (blue) and the NMR set (orange). (bottom) Relative cluster energies as a function of dihedral angle for the NMR set (orange) and random selections of the MD set (orange)

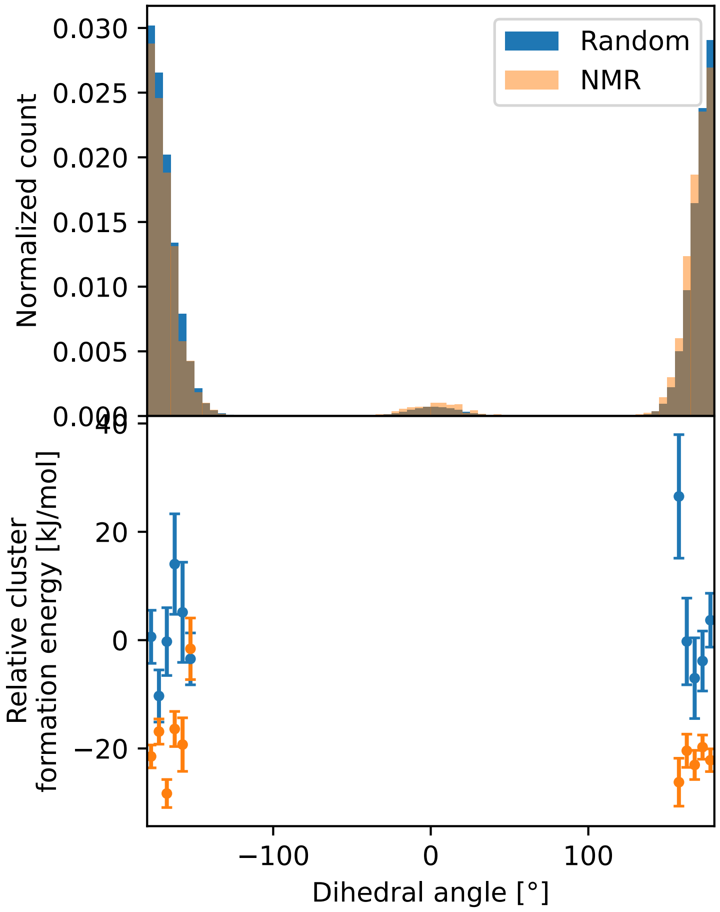

Figure S12: (top) histogram of torsion angle between C19-C20-N21-C22 for the MD set (blue) and the NMR set (orange). (bottom) Relative cluster energies as a function of dihedral angle for the NMR set (orange) and random selections of the MD set (orange)

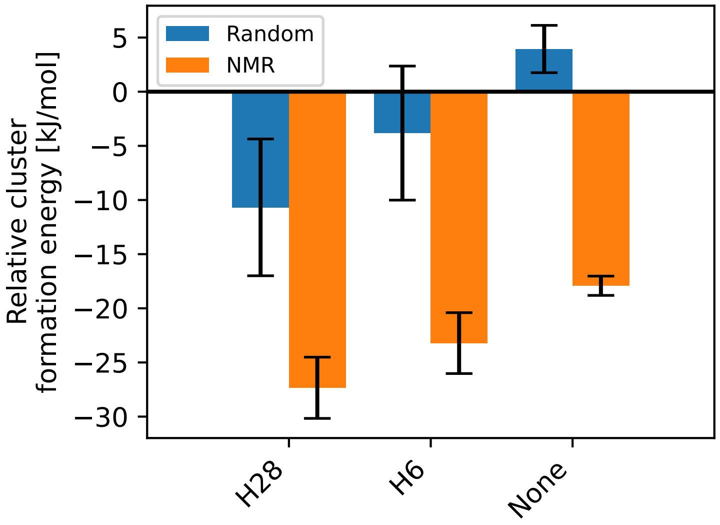

Figure S13: Average relative cluster energies for the hydrogen bond interaction with N5 for the MD set in (blue) and the NMR set (orange)

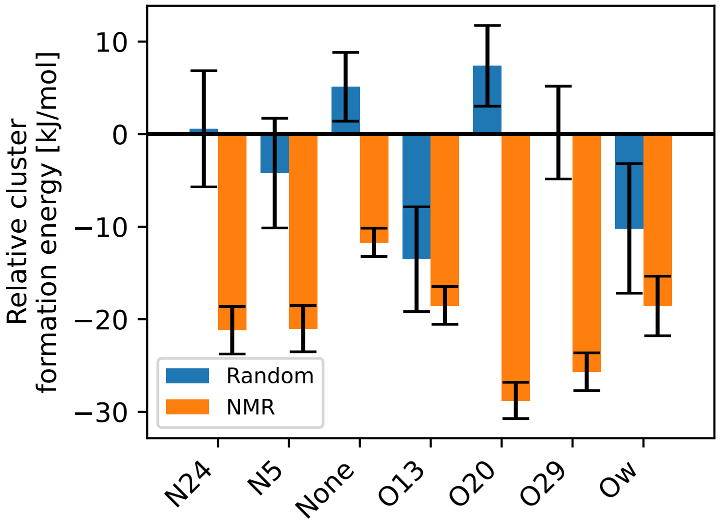

Figure S14: Average **r**elative cluster energies for the hydrogen bond interaction with N5 for the MD set in (blue) and the NMR set (orange)

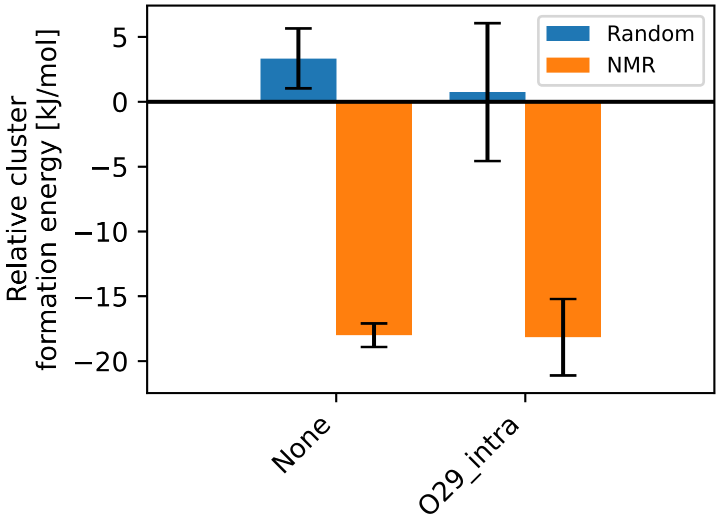

Figure S15: Average **r**elative cluster energies for the hydrogen bond interaction with N21 for the MD set in (blue) and the NMR set (orange)

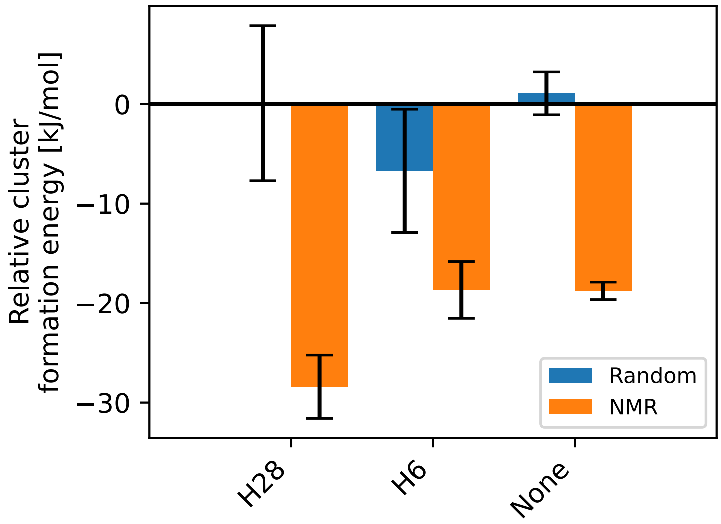

Figure S16: Average **r**elative cluster energies for the hydrogen bond interaction with N24 for the MD set in (blue) and the NMR set (orange)

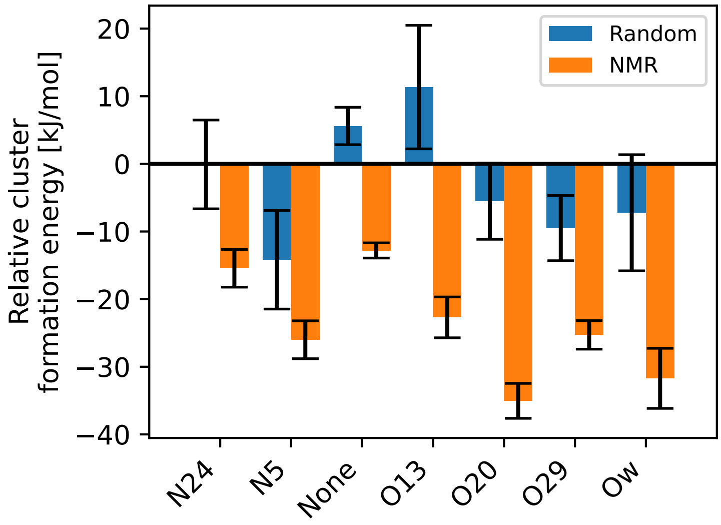

Figure S17: Average **r**elative cluster energies for the hydrogen bond interaction with N28 for the MD set in (blue) and the NMR set (orange)

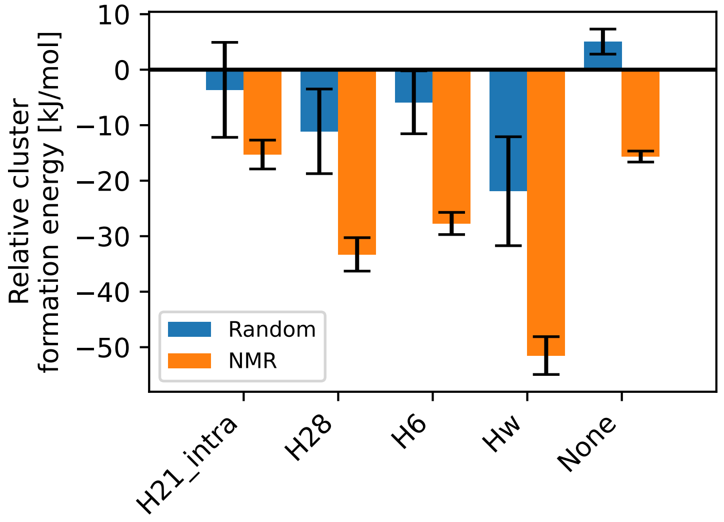

Figure S18: Average **r**elative cluster energies for the hydrogen bond interaction with O13 for the MD set in (blue) and the NMR set (orange)

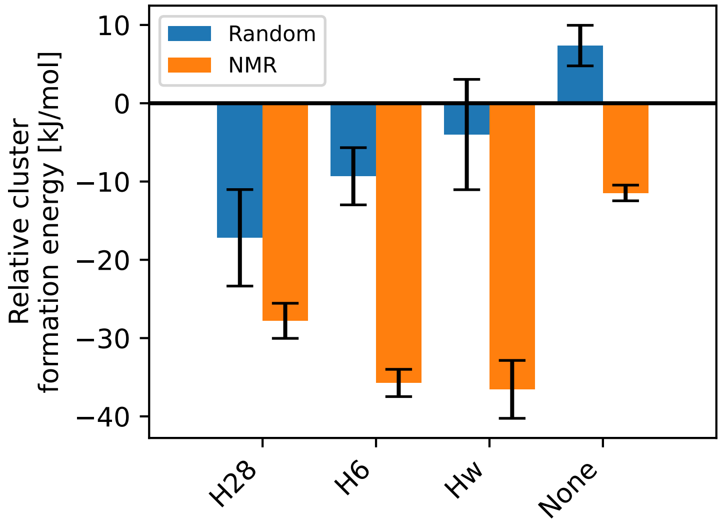

Figure S19: Average **r**elative cluster energies for the hydrogen bond interaction with O20 for the MD set in (blue) and the NMR set (orange)

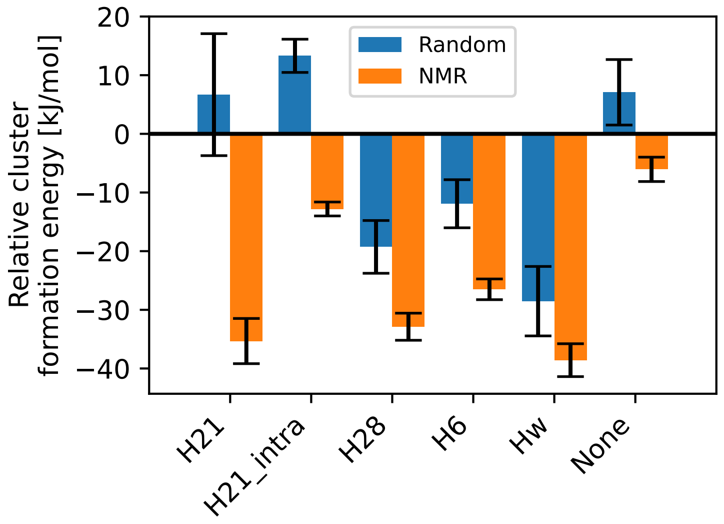

Figure S20: Average relative cluster energies for the hydrogen bond interaction with O29 for the MD set in (blue) and the NMR set (orange)

References

1 Cordova, M. *et al.* Structure determination of an amorphous drug through large-scale NMR predictions. *Nature Communications* **12**, 2964 (2021).

2 Becke, A. D. Density-Functional Thermochemistry .3. The Role of Exact Exchange. *J Chem Phys* **98**, 5648-5652 (1993). https://doi.org/Doi 10.1063/1.464913

3 Grimme, S., Antony, J., Ehrlich, S. & Krieg, H. A consistent and accurate ab initio parametrization of density functional dispersion correction (DFT-D) for the 94 elements H-Pu. *J Chem Phys* **132** (2010). https://doi.org/Artn 154104
Pmid 20423165
10.1063/1.3382344

4 Rassolov, V. A., Ratner, M. A., Pople, J. A., Redfern, P. C. & Curtiss, L. A. 6-31G*basis set for third-row atoms. *Journal of Computational Chemistry* **22**, 976-984 (2001). https://doi.org/DOI 10.1002/jcc.1058

5 Stephens, P. J., Devlin, F. J., Chabalowski, C. F. & Frisch, M. J. Ab-Initio Calculation of Vibrational Absorption and Circular-Dichroism Spectra Using Density-Functional Force-Fields. *J Phys Chem-Us* **98**, 11623-11627 (1994). https://doi.org/DOI 10.1021/j100096a001

6 Gaussian 09 v. Revision D.01 (Gaussian, Inc., Wallingford CT, 2016).

7 BIOVIA Materials Studio v. Release 2017 (BIOVIA, Dassault Systèmes, San Diego, 2017).

8 Sun, H. *et al.* COMPASS II: extended coverage for polymer and drug-like molecule databases. *J Mol Model* **22** (2016). https://doi.org/ARTN 47
10.1007/s00894-016-2909-0

9 Banks, J. L. *et al.* Integrated modeling program, applied chemical theory (IMPACT). *Journal of Computational Chemistry* **26**, 1752-1780 (2005). https://doi.org/10.1002/jcc.20292

10 Jorgensen, W. L., Maxwell, D. S. & TiradoRives, J. Development and testing of the OPLS all-atom force field on conformational energetics and properties of organic liquids. *Journal of the American Chemical Society* **118**, 11225-11236 (1996). https://doi.org/DOI 10.1021/ja9621760

11 ffld_server v. Release 2017-3 (Schrodinger, LLC, New York, NY, 2017).

12 Frolov, A. I. & Kiselev, M. G. Prediction of Cosolvent Effect on Solvation Free Energies and Solubilities of Organic Compounds in Supercritical Carbon Dioxide Based on Fully Atomistic Molecular Simulations. *J Phys Chem B* **118**, 11769-11780 (2014). https://doi.org/10.1021/jp505731z

13 Jorgensen, W. L., Chandrasekhar, J., Madura, J. D., Impey, R. W. & Klein, M. L. Comparison of Simple Potential Functions for Simulating Liquid Water. *J Chem Phys* **79**, 926-935 (1983). https://doi.org/Doi 10.1063/1.445869
